# Kidney Transplantation after Extended Multivisceral Resection for Pancreatic Ductal Adenocarcinoma

**DOI:** 10.1155/2018/3757382

**Published:** 2018-07-26

**Authors:** Hryhoriy Lapshyn, Louisa Bolm, Martin Nitschke, Andreas M. Luebke, Jakob R. Izbicki, Yogesh K. Vashist, Tobias Keck, Ulrich F. Wellner

**Affiliations:** ^1^Clinic for Surgery, University Clinic Schleswig-Holstein, Campus Lübeck, Lübeck, Germany; ^2^Department of Internal Medicine I, University Hospital of Schleswig-Holstein, Campus Lübeck, Lübeck, Germany; ^3^Institute of Pathology, University Medical Center Hamburg-Eppendorf, University of Hamburg, Hamburg, Germany; ^4^Department of General, Visceral and Thoracic Surgery, University Medical Center Hamburg-Eppendorf, University of Hamburg, Hamburg, Germany; ^5^Clinic for Visceral Surgery, Kantonsspital Aarau, Switzerland

## Abstract

Long-term survival in patients with pancreatic ductal adenocarcinoma (PDAC) is limited. Consequently, solid organ transplantation in PDAC patients is usually not considered. This is the first case report of kidney transplantation (KT) in a 57-year-old female patient after extended multivisceral resection for PDAC of the distal pancreas who had developed end-stage renal disease (ESRD) due to toxic kidney damage by chemotherapy. 13,5 years after initial PDAC-operation and 3 years after KT the patient remains in a good general health condition with sufficient function of the kidney allograft without local tumor recurrence or distant metastasis.

## 1. Introduction

Pancreatic ductal adenocarcinoma (PDAC) is an aggressive cancer with dismal prognosis and few therapeutic options. Median 5-year overall survival in patients with PDAC is limited to approximately 5% [1, 2]. PDAC is considered the most aggressive solid tumor characterized by early local and systemic tumor cell spread. Complete surgical resection remains the only curative option in PDAC; however, only few patients are eligible for this option. In patients undergoing combined surgical and oncologic treatment including curative resection, 5-year survival increases to 20-30% [3, 4]. As a consequence of dismal prognosis in PDAC patients, solid organ transplantation is usually not considered [5].

This is the first case report of kidney transplantation (KT) in a 57-year-old female patient after extended multivisceral resection for PDAC of the distal pancreas who had developed end-stage renal disease (ESRD) due to toxic kidney damage by adjuvant chemotherapy.

## 2. Case Presentation

The patient presented at the Department of Surgery, University Medical Center Hamburg-Eppendorf, in September 2004. At this point of time, the female patient was 46 years old and presented in a good clinical condition. She reported to suffer from progressive abdominal pain also affecting the back since June 2004. An abdominal computed tomography (CT) imaging disclosed a 5 x 4 cm solid mass of the pancreatic corpus/tail. A complete staging did not show distant metastases or pathologically enlarged lymph nodes. Endosonography confirmed these findings and additionally showed tumor infiltration into the stomach. A tumor biopsy revealed PDAC grade G2. Level of tumor marker carbohydrate antigen 19-9 (CA 19-9) was 2792 kU/l, significantly exceeding the reference range (normal value <37-40 kU/l). Initial renal function was normal.

**Figure 1:**
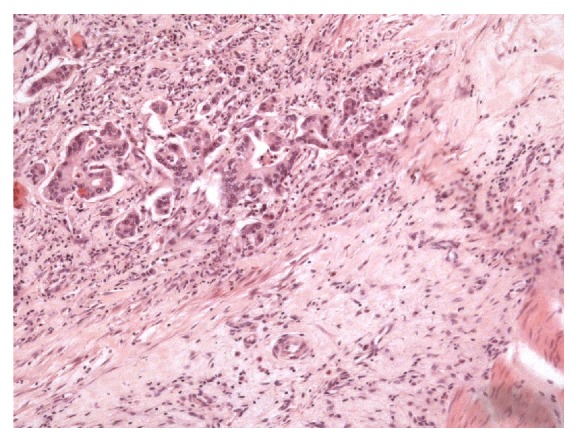
Pancreatic ductal adenocarcinoma with infiltrating atypical tubular and gland structures (hematoxylin and eosin staining (HE) 100x).

**Figure 2:**
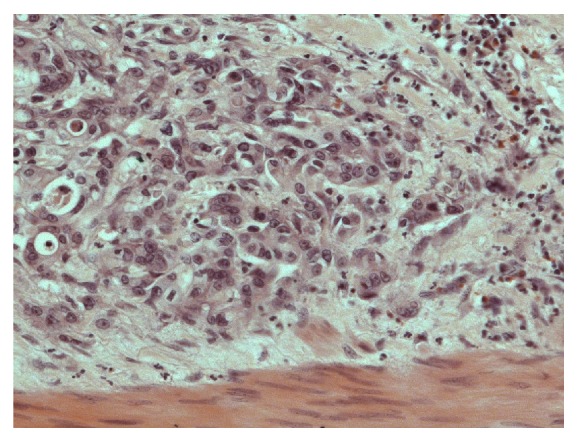
Pancreatic ductal adenocarcinoma with infiltrating atypical tubular and gland structures (hematoxylin and eosin staining (HE) 200x).

The tumor was considered resectable and the patient underwent radical extended pancreaticosplenectomy with adrenalectomy, subtotal gastrectomy, radical systematic lymphadenectomy, radical omentectomy, resection of the abdominal wall, tangential transverse colon resection, and cholecystectomy at the Department of Surgery, University Medical Center Hamburg-Eppendorf, in September 2004. Histopathological work-up revealed a pancreatic ductal adenocarcinoma (Figures 1 and 2) of the corpus with a diameter of 6.2 cm, tumor infiltration of the gastric and colon wall, localized peritoneal carcinomatosis, and lymph- and hemangiomas. Resection margins were negative. Final histopathological staging was pT3, pN1, pM1, L1, V1, G2-3, R0, and UICC-Stage IV. After the operation CA 19-9 increased to over 10000 kU/l.

Following complete resection, the patient received adjuvant chemotherapy with gemcitabine, followed by combined gemcitabine and cisplatin (November 2004 to April 2005) and combined gemcitabine together with mitomycin (five cycles April 2005 to January 2006) at standard dose. Chemotherapy was stopped after normalization of the tumor markers. In May 2006, the patient developed toxic chemotherapy-related end-stage renal disease (ESRD) requiring hemodialysis and a severe polyneuropathy.

Six years after resection the patient applied at the Transplant Center of the University Medical Center Hamburg-Eppendorf for KT. Regarding the initial aggressive tumor biology and the overall dismal prognosis of PDAC the patient was advised against the surgery.

In December 2012, the patient presented at the Transplant Center of the University of Lübeck (Germany) for KT. Due to PDAC history, the patient underwent extended follow-up examination. Abdominal magnetic resonance imaging (MRI) and positron emission tomography with deoxy-fluoro-D-glucose integrated with computed tomography (FDG-PET-CT), thoracic conventional X-ray, gastroscopy, colonoscopy, gynecological and other examinations, and measurement of tumor markers (carcinoembryonic antigen (CEA), CA 19-9) disclosed no evidence of local or systemic recurrence. The case was discussed at the interdisciplinary transplant conference of the Transplant Center Lübeck. KT was judged as feasible and indicated for this patient in an individual indication and strong patient motivation even after informed consent in this high-risk situation.

Allogenic KT was performed in March 2015 at the Transplantation Center at the University Medical Center Schleswig-Holstein, Lübeck. Cold ischemia time was 17 hours and warm ischemia time was 20 minutes. Intraoperatively, 6 para-iliac lymph nodes were removed. Histological work-up in fresh frozen section and terminal histology revealed reactive lymphadenopathy with no signs of malignancy.

Initial immunosuppression is comprised of Methylprednisolone, Basiliximab 40 mg, Mycophenolate-Mofetil, and Ciclosporin. Kidney function promptly recovered and ultrasonography showed sufficient perfusion of the transplanted kidney without evidence of urine retention.

Postoperatively, urinary tract infection and a small lymphocele at the upper pole and hilus of the allograft were diagnosed and managed conservatively. The patient was discharged on postoperative day 17.

Due to the patients history of malignancy a mechanistic target of rapamycin (mTOR) inhibitor based immunosuppression was considered but declined by the patient [6]. Currently, the patient presents in a good general health condition with sufficient function of the kidney transplant and a normal CA19-9. No local or distant PDAC recurrence has been detected in 33 months of follow-up.

A written informed consent was obtained from the patient for the publication of this case report, along with the corresponding figures.

## 3. Conclusion

This is the first case of KT after extended multivisceral resection for PDAC. Our data suggest KT as a feasible treatment in individual patients with history of aggressive malignant disease and posttreatment complete remission for many years as biological selection. The benefit of renal transplantation and quality of life may overweigh the risk of recurrent malignancy in this case and transplantation should not generally be excluded in selected cases with a history of malignant disease.
